# A *Drosophila* RNAi library modulates Hippo pathway-dependent tissue growth

**DOI:** 10.1038/ncomms10368

**Published:** 2016-01-13

**Authors:** Joseph H.A. Vissers, Samuel A. Manning, Aishwarya Kulkarni, Kieran F. Harvey

**Affiliations:** 1 Cell Growth and Proliferation Laboratory, Peter MacCallum Cancer Centre, 7 St Andrews Place, East Melbourne, Melbourne, Victoria 3002, Australia; 2 Sir Peter MacCallum Department of Oncology, University of Melbourne, Parkville, Victoria 3010, Australia; 3 Department of Pathology, University of Melbourne, Parkville, Victoria 3010, Australia

## Abstract

Libraries of transgenic *Drosophila melanogaster* carrying RNA interference (RNAi) constructs have been used extensively to perform large-scale functional genetic screens *in vivo*. For example, RNAi screens have facilitated the discovery of multiple components of the Hippo pathway, an evolutionarily conserved growth-regulatory network. Here we investigate an important technical limitation with the widely used VDRC KK RNAi collection. We find that approximately 25% of VDRC KK RNAi lines cause false-positive enhancement of the Hippo pathway, owing to ectopic expression of the Tiptop transcription factor. Of relevance to the broader *Drosophila* community, ectopic *tiptop (tio)* expression can also cause organ malformations and mask phenotypes such as organ overgrowth. To enhance the use of the VDRC KK RNAi library, we have generated a *D. melanogaster* strain that will allow researchers to test, in a single cross, whether their genetic screen of interest will be affected by ectopic *tio* expression.

## Abstract (web summary)

*Drosophila* RNAi libraries are commonly used to perform large-scale functional genetics screens *in vivo*. Here the authors find that a subset of lines from the VDRC KK RNAi line cause false-positive enhancement of the Hippo pathway, and provide a strain that can test whether a genetic screen of interest will be affected by this technical artefact.

## Graphical abstract

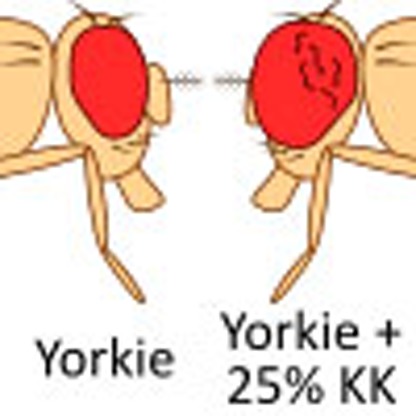

Genetic screens using the vinegar fly, *Drosophila melanogaster*, have been essential for our understanding of metazoan development. The generation of libraries of transgenic flies carrying inducible RNA interference (RNAi) vectors has enabled high-throughput functional genetic screens *in vivo*^1^. This technology has been successfully applied to uncover new regulators of a diverse range of biological functions^2^,^3^.

The VDRC KK collection is an RNAi library that is used in *Drosophila* laboratories globally (www.vdrc.at). It was designed to feature site-specific integration of hairpin RNAs carried in a transgenic vector at a defined locus (40D on chromosome 2L), to ensure consistent expression of different RNA hairpins and to alleviate the potential for disruption or misexpression of host genes. However, a recent report showed that only a subset of VDRC KK RNAi lines actually carry a transgene at 40D, whereas all lines possess a transgene at a secondary site that was unintentionally targeted (30B on chromosome 2L)^4^. Importantly, transgene integration at 40D is associated with dominant phenotypes when crossed to some *Gal4* drivers^4^.

The Hippo pathway constitutes an essential and evolutionarily conserved growth-regulatory network^5^,^6^,^7^,^8^,^9^,^10^. RNAi screens in *D. melanogaster* have facilitated the discovery of many new components of the Hippo pathway^11^,^12^,^13^,^14^,^15^,^16^,^17^. The key downstream component of the Hippo pathway is Yorkie (Yki), a growth-promoting transcriptional co-activator^18^. Expression of an activated form of Yki (Yki^S168A^) in the eyes leads to overgrowth, which we and others have used as a sensitized background to screen for new Hippo pathway proteins^11^,^12^,^13^,^14^.

In this study, we show that transgene insertion at the 40D locus in a subset of VDRC KK RNAi lines is associated with aberrant enhancement of Hippo pathway phenotypes. The effect is not due to knockdown of intended target genes by RNAi. Rather, our data suggest that the effect is due to ectopic UAS-driven expression of the Tiptop transcription factor. Ectopic *tiptop (tio)* expression linked to a subset of VDRC KK RNAi lines causes deleterious phenotypes in different organs, as evidenced by the fact that broad expression causes organism lethality^4^. Of relevance to the *Drosophila* research community, we describe a *D. melanogaster* strain that will allow researchers to test, in a single cross, whether their genetic screen of interest will be affected by ectopic *tio* expression.

## Results and Discussion

### 25% of VDRC KK RNAi lines enhance Yki-mediated overgrowth

**Figure 1:**
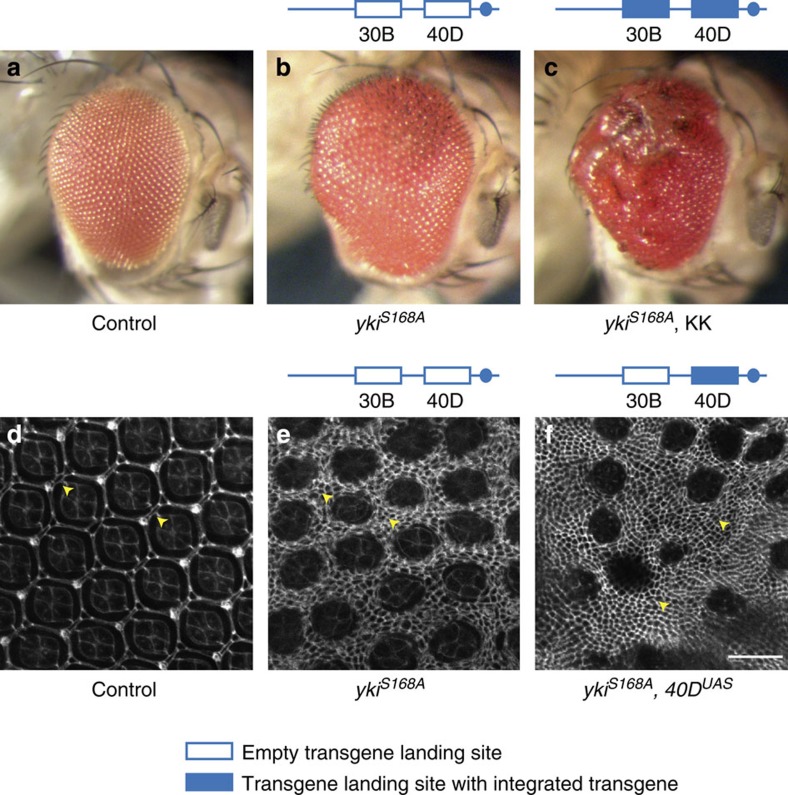
Select VDRC KK RNAi lines enhance Yorkie-induced eye overgrowth. (**a**) Wildtype eyes (F_1_ flies of VDRC genetic background crossed to *GMR-Gal4, UAS-EYFP*). (**b**,**c**) Adult eye phenotypes of F_1_ flies carrying *GMR-Gal4, UAS-yki*^*S168A*^*–YFP* transgenes crossed to VDRC genetic background (**b**) or VDRC line 105838 (targeting *Caf1*) (**c**). (**d**–**f**) Posterior area of pupal eyes of wildtype (**d**) and F_1_ flies carrying *GMR-Gal4, UAS-yki*^*S168A*^*–YFP* transgenes crossed to VDRC genetic background (**e**) or a recombinant harbouring Gal4-responsive UAS repeats but no functional shRNA-coding sequence at 40D (‘*40D*^*UAS*^') (**f**). Pupal eyes were fixed and stained with Discs large antibody to highlight individual cells. Yellow arrowheads indicate interommatidial cells. Scale bar, 20 μm.

To screen for new components of the Hippo pathway, we drove expression of an activated form of Yki (Yki^S168A^)^19^ in the eye using *GMR-Gal4* and crossed this strain to RNAi lines from the VDRC KK library. We then scored for modulation of the Yki-induced eye overgrowth phenotype. We observed that a large number of VDRC KK RNAi lines enhanced Yki-induced eye overgrowth, particularly in the posterior part of the eye, which we scored as enhancers (Fig. 1a–c). However, in experiments analysing a subset of these enhancers with independent RNAi lines (such as VDRC GD, TRiP or NIG RNAi lines) or mutant alleles, the original phenotype was not recapitulated (Supplementary Fig. 1), suggesting that it was not caused by depletion of the intended target gene.

**Figure 2:**
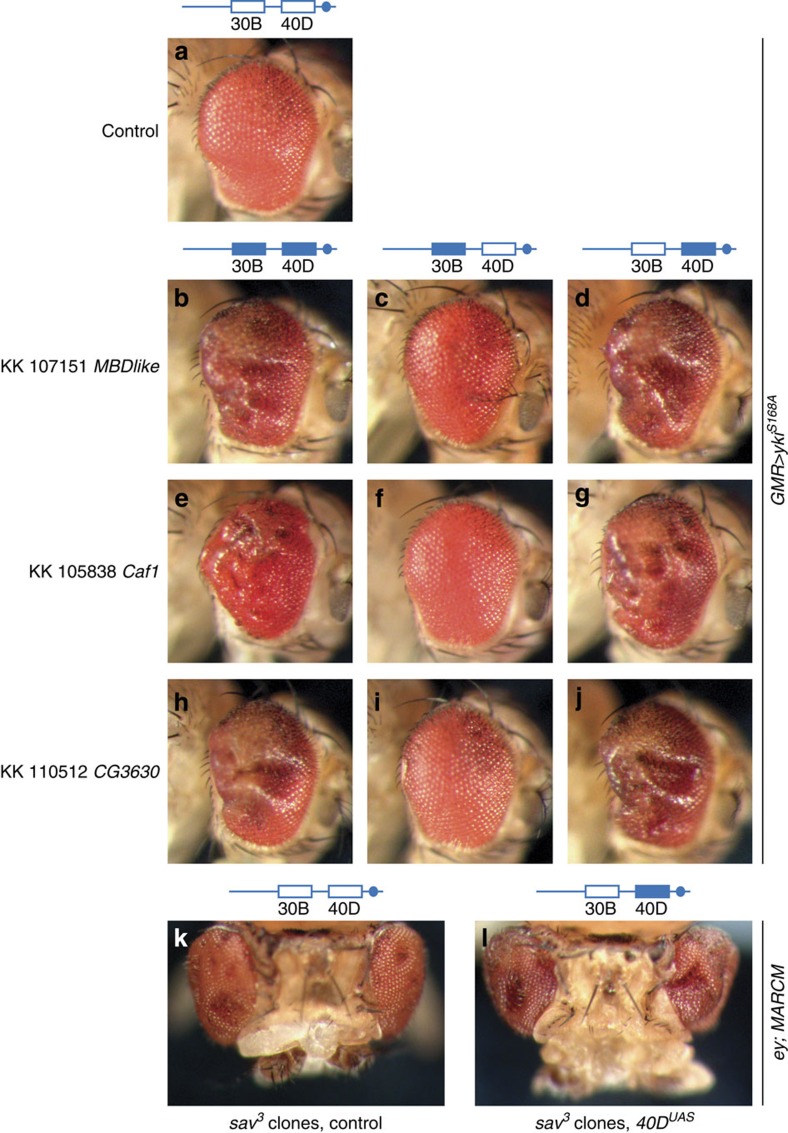
Enhancement of Yorkie-induced eye overgrowth by VDRC KK RNAi lines co-segregates with insertion at 40D and is independent of knockdown of intended target genes. (**a**–**j**) Adult eye phenotypes of F_1_ flies carrying *GMR-Gal4, UAS-yki*^*S168A*^*–YFP* transgenes crossed to VDRC genetic background (**a**), VDRC line 107151 (*MBD-like*) with double insertion (**b**), single insertion at 30B (**c**) and 40D (**d**), VDRC line 105838 (*Caf1*) with double insertion (**e**), single insertion at 30B (**f**) and 40D (**g**), VDRC line 110512 (*CG3630*) with double insertion (**h**), single insertion at 30B (**i**) and 40D. The transgene insertion at 40D in line 110512 does not carry a functional hairpin-coding sequence, hence it is called ‘*40D*^*UAS*^' throughout this manuscript. (**j**). (**k**,**l**) Eye phenotypes of flies harbouring *sav*^*3*^ clones expressing Gal4 in the eye combined with the VDRC genetic background (**k**) and *40D*^*UAS*^ (**l**). Sav is an upstream negative regulator of Yki, therefore the *sav*^*3*^ mutation causes Yki hyperactivation. Clones were generated using the Mosaic analysis with a repressible cell marker (MARCM) system under the control of the *eyeless* (*ey*) promoter.

Based on the findings of Green *et al*.^4^, we genotyped these KK RNAi lines by PCR to determine their integration site occupancy. We observed that most KK RNAi lines that enhanced Yki-driven eye overgrowth possessed transgenes at both 40D and 30B on chromosome arm 2L (Fig. 2a–g). Notably, the percentage of KK RNAi lines we scored as enhancers (38 out of 150, 25%) is very similar to the percentage of KK RNAi lines reported by Green *et al*.^4^, to contain insertions at both 40D and 30B (9/39, 23%). We separated the two insertion sites in these lines by meiotic recombination and observed that enhancement of Yki-mediated eye overgrowth co-segregated with transgene insertion at 40D (Fig. 2a–g). This result strongly suggests that the observed phenotypic enhancement is an artefact associated with transgene insertion at the 40D locus. However, it did not formally exclude the possible contribution of knockdown of intended target genes.

To investigate this further, we made use of a KK RNAi line that harbours Gal4-responsive UAS repeats but no functional short hairpin RNA (shRNA) coding sequence at 40D (E. Green, personal communication, see also Methods). We used meiotic recombination to remove the transgene insertion at 30B. Using the resulting ‘*40D*^*UAS*^' line, we tested whether enhancement of Yki-mediated overgrowth was caused by UAS-dependent overexpression of a gene at 40D or expression of the shRNA. The *40D*^*UAS*^ line enhanced Yki-mediated overgrowth (Fig. 2j) and caused a substantial increase in interommatidial cells (Fig. 1f), considered a classic Hippo pathway mutant phenotype^20^.

By contrast, in three independent lines expressing shRNA hairpins for *MBD-like*, *Caf1* and *CG3630* from the insertion site at 30B, but not 40D, we observed no modification of Yki-driven eye overgrowth (Fig. 2a–j). Therefore, enhancement of Yki-mediated eye overgrowth was caused by transgene integration at 40D, rather than RNAi-mediated depletion of target genes.

To assess this result in an independent genetic context, we generated flies with clones of eye tissue mutant for *salvador (sav)*, a member of the core kinase cassette of the Hippo pathway^20^,^21^. Loss of function of *sav* leads to Yki hyperactivation^18^ and overgrowth^20^,^21^. The eye overgrowth phenotype of flies harbouring *sav* mutant clones expressing *Gal4* combined with *40D*^*UAS*^ was enhanced to a similar degree as we observed when *Yki*^*S168A*^ was overexpressed (Fig. 2k,l). This confirms that transgene insertion at 40D in KK RNAi lines enhances Yki-mediated tissue overgrowth irrespective of the mechanism by which Yki is activated.

### Ectopic tiptop expression in a subset of VDRC KK RNAi lines

**Figure 3:**
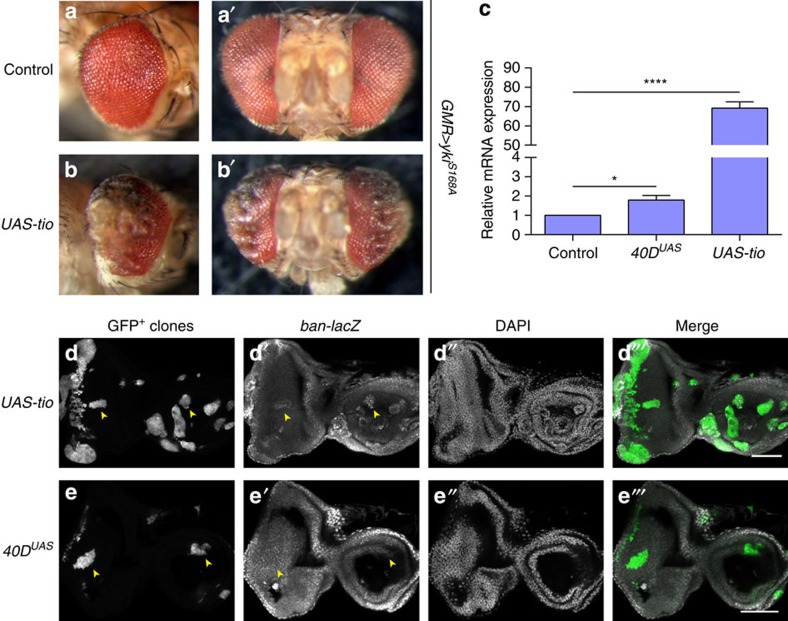
Overexpression of *tiptop* enhances Yorkie-induced eye overgrowth and induces bantam expression. (**a**,**b**) Adult eye phenotypes of F_1_ flies carrying *GMR-Gal4, UAS-yki*^*S168A*^*–YFP* transgenes crossed to *UAS* control (**a**) and *UAS-tio* (**b**). (**c**) *Tio* mRNA expression in eye imaginal discs of F_1_ flies carrying *GMR-Gal4* crossed to VDRC genetic background, *40D*^*UAS*^ and *UAS-tio* (positive control). QPCR was performed using two different primer sets probing *tio* mRNA expression, and normalized against *Act5c* and *Rp49* mRNA-loading controls, yielding identical results (only *tio* primer set 1 and *Act5c* results shown). Average results from three independent experiments are shown. Error bars indicate s.e.m. *P<0.05, ****P<0.0001, unpaired two-tailed *t*-test. (**d**,**e**) Actin-Flp-out clones (yellow arrowheads) expressing *Gal4* in combination with *UAS-tio* (**d**) or *40D*^*UAS*^ (**e**) in larval eye imaginal discs expressing the *ban-lacZ* reporter. Scale bars, 50 μm.

Green *et al*.^4^ suggested that the dominant effects caused by transgene insertion at 40D were caused by overexpression of the *tio* gene, given that KK RNAi lines were engineered to be inserted at a locus at 40D that is located in the *tio* 5′ untranslated region. This hypothesis gained our attention, because Yki was reported to regulate eye growth, at least in part, together with Teashirt (Tsh)^22^, a functionally redundant *tio* paralogue^23^. Indeed, an independent *UAS*-regulated *tio* transgene^24^ also enhanced Yki-mediated eye overgrowth (Fig. 3a,b). Moreover, *tio* mRNA expression was increased approximately twofold in eye imaginal discs from larvae combining eye-specific Gal4 expression with the aforementioned *40D*^*UAS*^ line (Fig. 3c). Taken together, these findings strongly suggest that ectopic *tio* expression caused by insertion of *UAS*-carrying transgenes at 40D in some KK RNAi lines is responsible for enhancement of Yki-mediated eye overgrowth. The *40D*^*UAS*^ line or *UAS-tio* did not enhance eye growth in flies expressing EYFP (control), dMyc or insulin receptor (InR), although high *tio* overexpression caused patterning defects in the posterior part of the eye (Supplementary Fig. 2). Therefore, ectopic *tio* expression specifically modulates Hippo pathway-mediated tissue growth, at least when these growth pathways are modulated in the developing eye using *GMR-Gal4*.

An important validation of newly identified Hippo pathway components is the demonstration that they regulate expression of Yki target genes such as *bantam (ban)*. We found that, although high *tio* overexpression induced *ban* expression, the *40D*^*UAS*^ line did not (Fig. 3d,e). This shows that, like *tsh*, ectopic *tio* expression can drive expression of Yki target genes. Changes in *ban* expression due to ectopic *tio* expression in KK lines were probably beyond the limits of detection in our experimental setting given that we observed clear effects on tissue growth.

### Resources to enhance the use of the VDRC KK RNAi library

**Figure 4:**
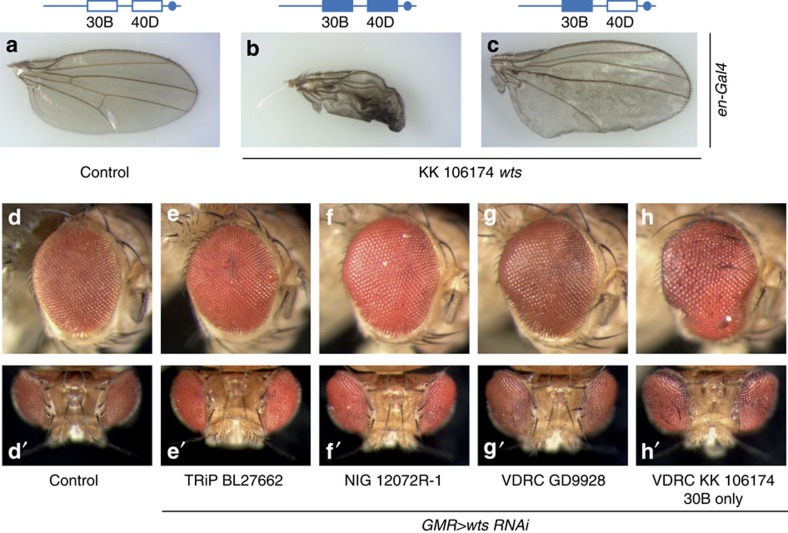
Separation of insertion sites in the Warts KK RNAi line reveals potential for false-negative screening results and shows the power of KK RNAi lines if the 40D integration is removed. (**a**–**c**) Wings of control flies (**a**) and F_1_ flies carrying *en-Gal4* crossed to VDRC line 106174 (*wts*) with double insertion (**b**) or single insertion at 30B (**c**). Flies were reared at 18 °C. (**d**–**h**) Adult eye phenotypes of F_1_ flies carrying *GMR-Gal4* crossed to VDRC genetic background (**d**), and *wts* RNAi lines Bloomington TRiP 27662 (**e**), NIG 12072R-1 (**f**), VDRC GD line 9928 (**g**) and VDRC KK line 106174, insertion at 30B only (**h**).

To provide a resource for other researchers, we genotyped a subset of commonly used KK RNAi lines directed against Hippo pathway genes and identified several that have transgene insertions at 40D (Supplementary Table 1 and Supplementary Fig. 3). Separation of insertion sites of the KK RNAi line targeting the Hippo pathway kinase Warts (Wts) revealed two important findings: first, wing overgrowth caused by Wts knockdown was masked by the non-inflating wing phenotype caused by transgene insertion at 40D^4^ (Fig. 4a–c); second, eye-specific Wts knockdown in a line that harbours a transgene insertion at 30B only, led to more dramatic eye overgrowth than other publicly available Wts RNAi lines (Fig. 4d–h), illustrating the potential power of KK RNAi lines when free of transgene insertions at 40D.

Finally, we crossed the *40D*^*UAS*^ line to a collection of Gal4 drivers and assessed overt phenotypes in adult progeny (Supplementary Table 2). In particular, crosses to wing drivers caused substantial malformations, whereas eye drivers did not. Some wing drivers led to the non-inflating wing phenotype as described for *elav-gal4*^*155*^ (ref. 4) (Supplementary Fig. 4b), whereas others caused wing shape changes (Supplementary Fig. 4d). Broad expression of the *40D*^*UAS*^ line caused organism lethality.

In conclusion, some KK RNAi lines can give false-positive results in eye-based screens for Hippo pathway genes and can also cloud growth screens performed in tissues such as the wing. The VDRC KK RNAi library has proven to be an extremely powerful resource for exploring gene function. Importantly, the *40D*^*UAS*^ flies we have generated will allow researchers to test, in a single cross (Supplementary Fig. 4e), whether their screen of interest will be affected by ectopic *tio* expression that is associated with a subset of KK RNAi lines.

## Methods

### *Drosophila* genetics

VDRC KK and GD RNAi lines were from VDRC, Austria. Other RNAi lines were from NIG, Japan or Bloomington, USA. *Drosophila* KK RNAi strains carrying a single transgene insertion were generated by meiotic recombination and their genotype confirmed by PCR^4^.

Male flies from the KK RNAi library, recombinant strains and *w;; UAS-tio*^24^ were crossed to *w;; GMR-Gal4, UAS-yki*^*S168A*^*–YFP/TM6B*^11^*, w;; GMR-Gal4, UAS-EYFP/TM6B, w;; GMR-Gal4* or *w; en-Gal4* virgin female flies.

For the eyFlp, MARCM experiment, genotypes were:

*y w eyFlp, UAS–green fluorescent protein (UAS–GFP); GD60100/+; tub-Gal4 FRT82B tub-Gal80/FRT82B sav^3^*

*y w eyFlp, UAS–GFP; 40D^UAS^/+; tub-Gal4 FRT82B tub-Gal80/FRT82B sav^3^*

For the Flp-out experiment, genotypes were:

*y w hsFLP;40DUAS/+; act>CD2>GAL4, UAS–GFP/ ban-lacZ*

*y w hsFLP;;act>CD2>GAL4, UAS–GFP/ UAS-tio-FL, ban-lacZ*

The *ban-lacZ* allele is described in Flybase (*P{lacW}banL1170a*) and was from Bloomington.

At least 30 adult organs (eyes and wings) of 1–2-day-old females were assessed.

See Supplementary Methods for genotypes presented in Supplementary Information.

### PCR and sequencing

Primers were designed using Primer3. Genomic DNA was isolated by crushing flies in lysis buffer (0.2 M Sucrose, 0.1 M Tris, pH 9.2, 50 mM EDTA, 0.5% SDS), 10 min incubation at 65 °C, addition of 1 M KAc, 15 min incubation on ice, phenol–chloroform extraction and isopropanol precipitation. KK insertion site occupancy was determined as in ref. 4. Briefly, occupancy of the transgene insertion site at 40D was determined in multiplex PCR using the following primers:

C_Genomic_F 5′-GCCCACTGTCAGCTCTCAAC-3′

pKC26_R 5′-TGTAAAACGACGGCCAGT-3′

pKC43_R 5′-TCGCTCGTTGCAGAATAGTCC-3′

PCR using these primers yields a ∼450 bp product in the case of an insertion, or a ∼1,050 bp product in the case of an empty insertion site, at 40D.

For the insertion at 30B, the pKC26_R and pKC43_R primers were multiplexed with the following primer:

NC_Genomic_F 5′-GCTGGCGAACTGTCAATCAC-3′

PCR using these primers results in a ∼600 bp product in the case of an insertion, and a ∼1,200 bp product in the case of an empty insertion site, at 30B.

PCR was performed using Qiagen Taq PCR master mix in an Eppendorf thermal cycler with the following programme: 95 °C, 120 s initial denaturation, 30 cycle reaction (95 °C 15 s denaturation, 51 °C 15 s annealing, 72 °C 80 s extension), and a final 72 °C, 120 s extension. Primers to detect whether pKC26 was filled were:

KK_ UAS_f 5′-TAGGCACCCCAGGCTTTAC-3′

KK_UAS_r 5′-TTTGTCCAATTATGTCACACCAC-3′

PCR was performed with the following programme: 95 °C, 120 s initial denaturation, 30 cycle reaction (95 °C 15 s denaturation, 50 °C 15 s annealing, 72 °C 45 s extension), and a final 72 °C, 120 s extension. These primers amplify a 700 bp product within empty pKC26, and do not amplify if a RNA hairpin-encoding sequence is inserted. PCR was performed using KK transformant ID 110512 genomic DNA, and the product cloned and sequenced. The sequence inserted is 5′-TAGGTCTTTCCCTTGGCGGGGCTTGACCTATCTAGA-3′. Blast searches using this sequence against *Drosophila* RefSeq RNA, genome and the nr database did not recover any meaningful matches.

### Quantitative real-time PCR

Per condition, 30 eye-antennal discs were dissected from late L3 larvae from crosses of *w;; GMR-Gal4* females to males of the indicated genotypes, and lysed in Trizol reagent (Life Technologies). RNA isolation was performed according to manufacturer's instructions. RNA (0.5 μg) was treated with DNAse (Promega) followed by addition of oligo-dT and retrotranscription using SuperScript III (Promega). Quantitative PCR was performed using Sybr Green Fast (Life Technologies) on a StepOnePlus system (Life Technologies). Quantitative PCR was performed using two different primer sets probing *tio* mRNA expression, and normalized against *Act5c* and *Rp49* mRNA-loading controls, yielding identical results (only *tio* primer set 1 and *Act5c* results shown).

The following primers were used:

qTio_fwd1_2430 5′-TGGAGAACAGCGGCAATAAC-3′

qTio_rev1_2557 5′-ATCCGTGGTCTCGCACAG-3′

qTio_fwd2_2388 5′-GTCTACTGGCGTTGAACTCC-3′

qTio_rev2_2529 5′-GCAGCTAGAGGATGGGCC-3′

qActin_5c_f 5′-CTCCAAGCAGGAGTACGACGAG-3′

qActin_5c_r 5′-CTTCCACCACTCGCACTTGC-3′

qRpL32_f 5′-CGCACCAAGCACTTCATCCG-3′

qRpL32_r 5′-GCACGTTGTGCACCAGGAAC-3′

### Microscopy

Pupal eyes were dissected in PBS from staged pupae 46 h after puparium formation. Flp-out clones were generated by 30 min of heat shock (37 °C), 48 h prior to dissection. L3 larvae were dissected in PBS. Samples were fixed for 20 min in 4% paraformaldehyde (Electron Microscopy Sciences #15710), rinsed once and washed three times for 10 min in 0.3% PBT. Samples were then blocked for 30 min in 0.1% PBT with 10% NGS and incubated for 4 h at 4 °C with primary antibodies in 0.1% PBT with 10% NGS. Samples were then washed three times for 12 min in 0.3% PBT, before secondary antibodies were added in 0.1% PBT and 10% NGS and incubated overnight at 4 °C. Samples were then washed three times for 12 min in 0.3% PBT, DAPI was added with the second wash. Samples were mounted in 90% glycerol in PBS and imaged. Confocal images were acquired using a Nikon C2 confocal microscope using a × 20 (numerical aperture (NA)=0.75) or 40 × (NA=1.00, oil immersion) objective and processed using Fiji (ImageJ).

Primary antibodies used were β-galactosidase (mouse, 1:100, Sigma G4644) and Discs large (mouse, 1:50, Developmental Studies Hybridoma Bank #2A1). Secondary antibody was anti-mouse 555 (goat, 1:500, Molecular Probes).

### Statistics

No statistical test was used to predetermine sample size in any of the experiments. For animal experiments, at least 30 animals were assessed in each experiment. No samples or animals were excluded from the analysis in any of the experiments. The experiments were not randomized. Investigators were not blinded to allocation during experiments or outcome assessment.

In Fig. 3c, an unpaired two-tailed *t*-test was used. The data met the assumptions of the test. S.e.m. is indicated. The variance is similar between the groups that are being statistically compared.

## Additional information

**How to cite this article:** Vissers, J. H. A. *et al*. A *Drosophila* RNAi library modulates Hippo pathway-dependent tissue growth. *Nat. Commun.* 7:10368 doi: 10.1038/ncomms10368 (2016).

## Supplementary Material

Supplementary Information Supplementary Figures 1-4, Supplementary Tables 1-2, Supplementary Methods and Supplementary References
