# Supplementary material for: A Drosophila RNAi library modulates Hippo pathway-dependent tissue growth
Source: Nat Commun. 2016 Jan 13;7:10368. doi: 10.1038/ncomms10368 (PMC4735554; doi:10.1038/ncomms10368)
Supplement: Supplementary Information — Supplementary Figures 1-4, Supplementary Tables 1-2, Supplementary Methods and Supplementary References [file ncomms10368-s1.pdf]

SUPPLEMENTARY FIGURES

Supplementary Figure 1

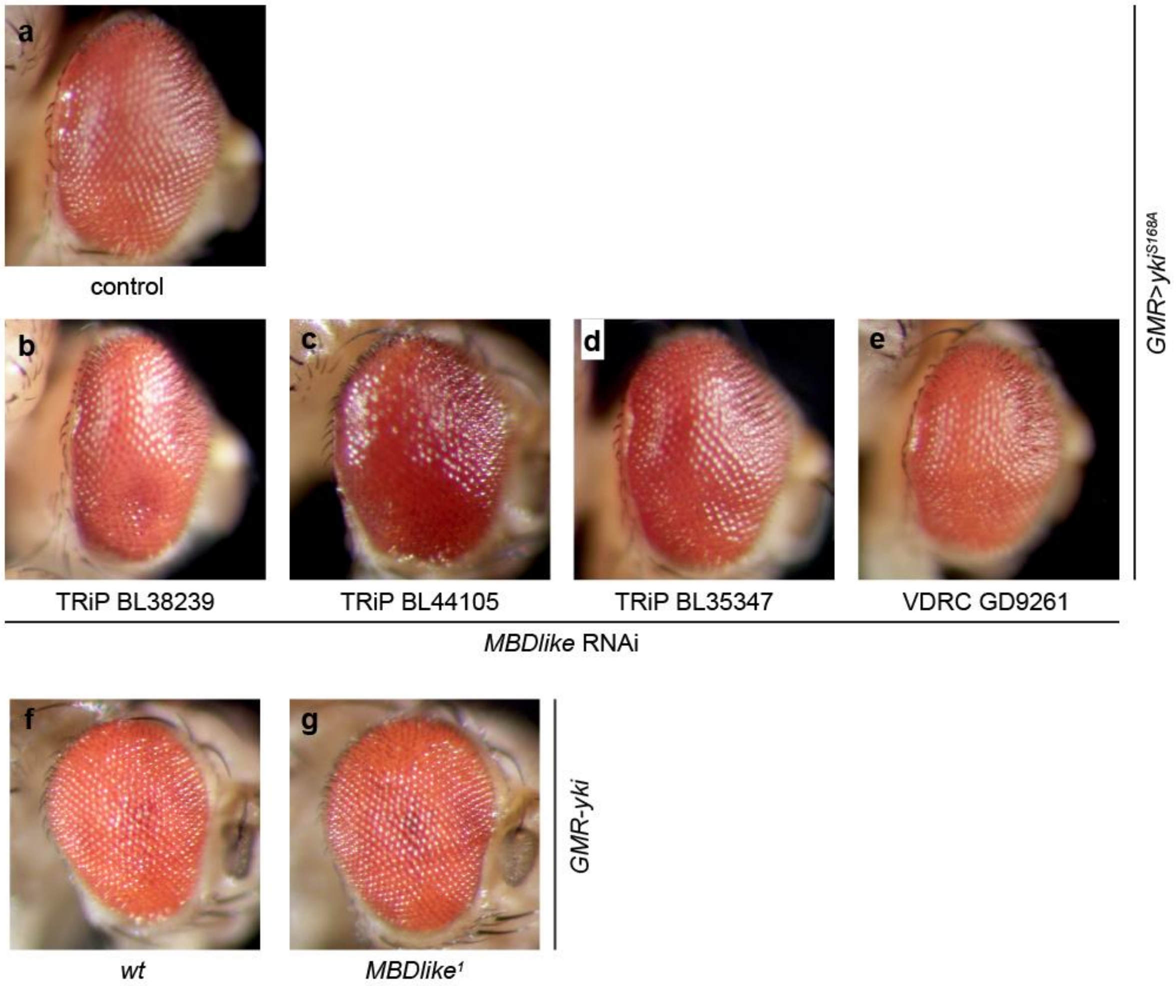

Supplementary Figure 1. Neither knockdown using alternative RNAi lines, nor mutation of *MBDlike* enhances the Yki eye-specific overexpression phenotype.

(a-e) Adult eye phenotypes of F_1 flies carrying *GMR-Gal4, UAS-Yki*^{S168A} transgenes crossed to a control RNAi line (β *galactosidase* RNAi VDRC GD51446) **(a)**, and *MBDlike* RNAi lines Bloomington TRiP 38239 **(b)**, 44105 **(c)**, 35347 **(d)** and VDRC GD9261 **(e)**.

(f,g) Adult eye phenotypes of *MBDlike* wild type **(f)** or mutant **(g)** flies carrying a *GMR-yki* transgene.

Supplementary Figure 2

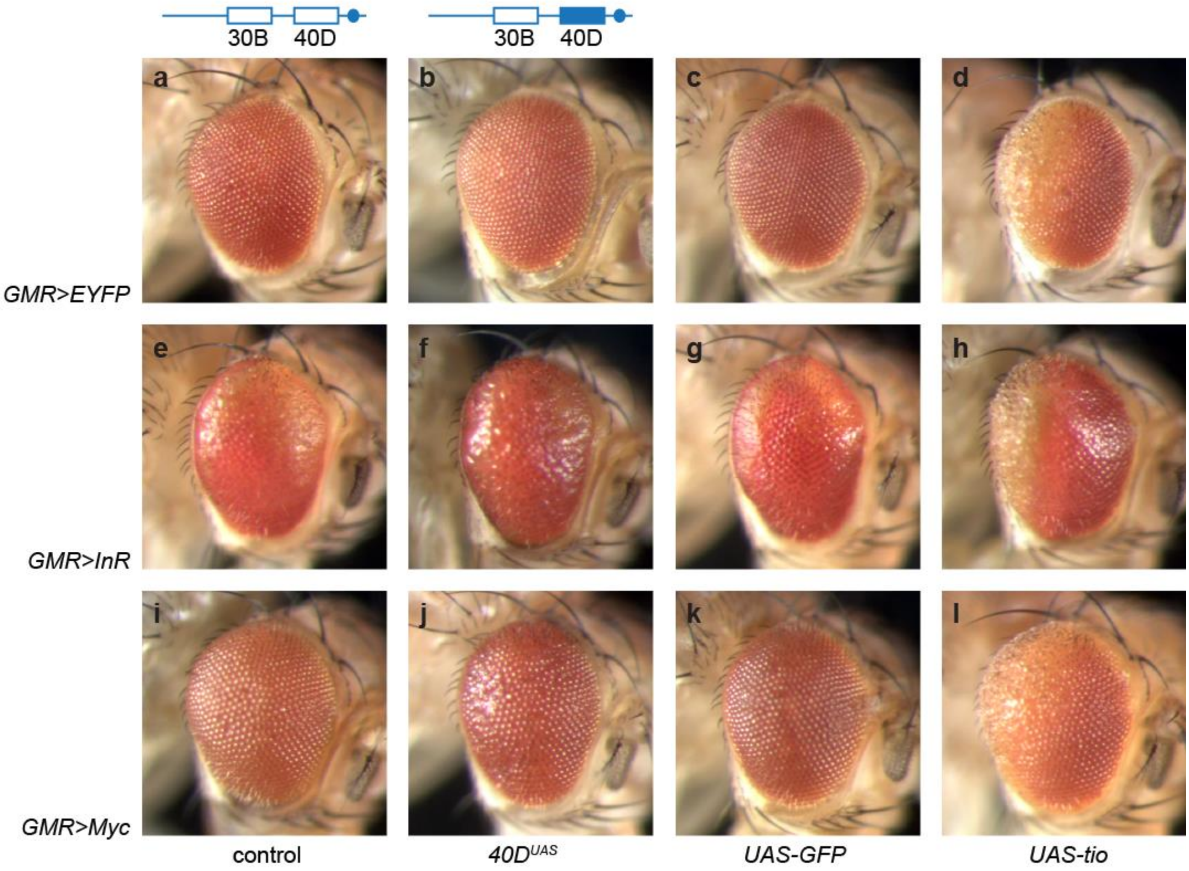

Supplementary Figure 2. Neither transgene insertion at 40D, nor *tiptop* overexpression enhances *dMyc* and *InR* eye-specific overexpression phenotypes.

Adult eye phenotypes of F_1 flies carrying *GMR-Gal4* and either *UAS-EYFP* **(a-d)**, *UAS-InR* **(e-h)**, or *UAS-dMyc* **(i-l)** transgenes crossed to; VDRC genetic background **(a, e, i)**, *40D*^{UAS} **(b, f, j)**, *UAS-GFP* **(c, g, k)**, *UAS-tio* **(d, h, l).**

Supplementary Figure 3

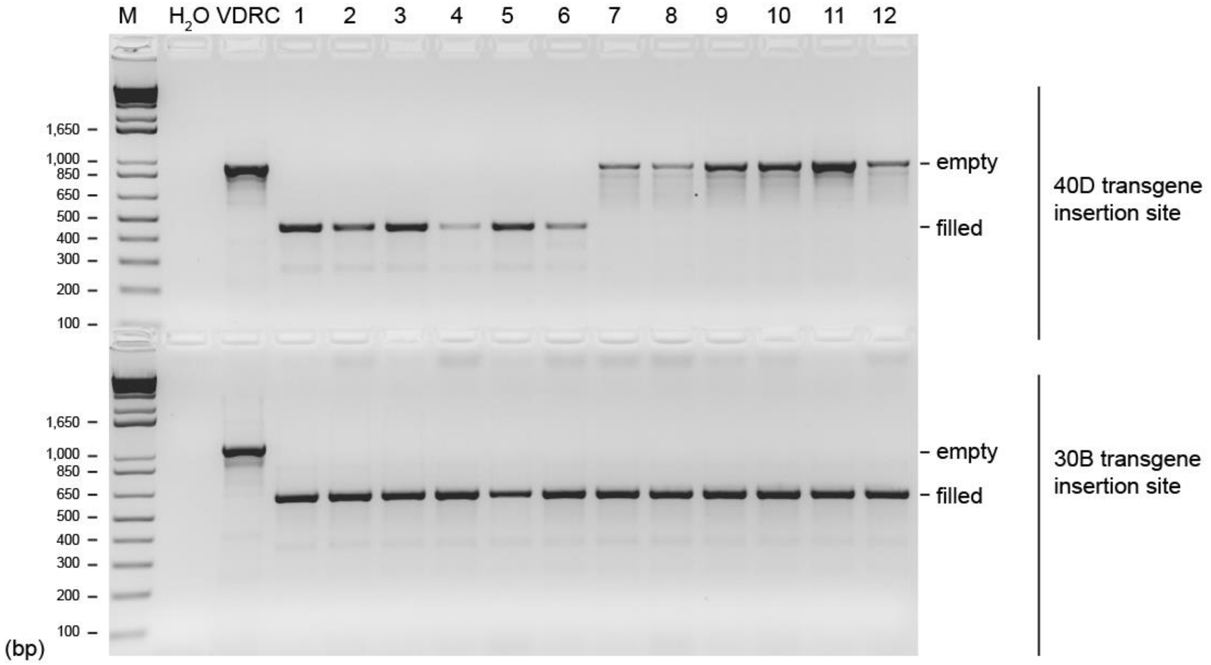

Supplementary Figure 3. Analysis of site occupancy in KK RNAi lines targeting known Hippo pathway components.

PCRs were performed as described in Methods[1] and run on 2% agarose gel. Numbering according to Supplementary Table 1. H_2O: water control. VDRC: VDRC genetic background.

Supplementary Figure 4

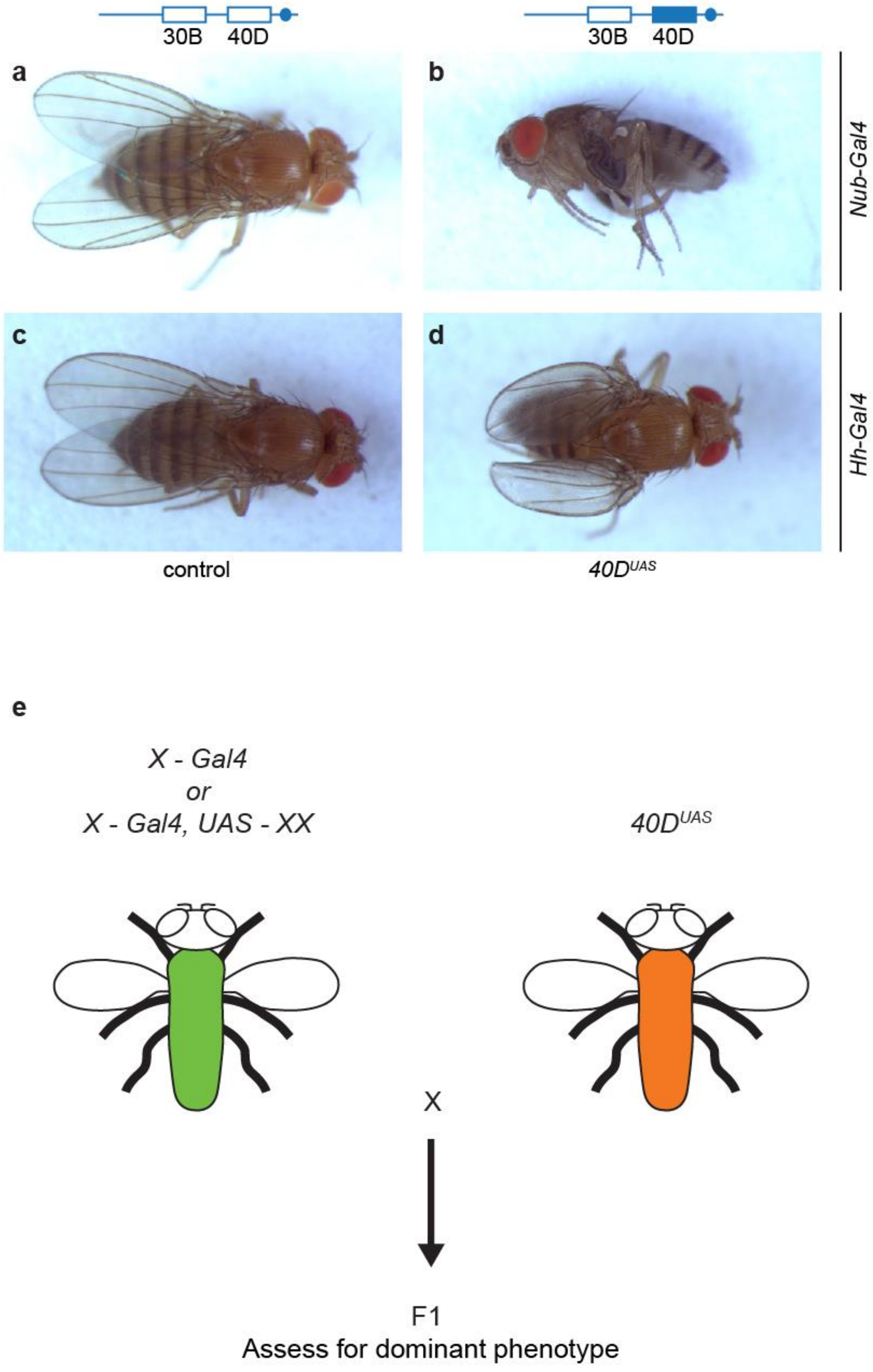

Supplementary Figure 4. The *40D*^{UAS} line can be used to determine whether screening system is affected by transgene integration at 40D in VDRC KK RNAi lines.

(a,b) F1 flies carrying *Nubbin-Gal4* and VDRC genetic background (control) **(a)** or *40D*^{UAS} **(b)**.

(c,d) F1 flies carrying *Hedgehog-Gal4* and VDRC genetic background (control) **(c)** or *40D*^{UAS} **(d)**.

(e) A typical screening line will harbour a *Gal4* driver of interest, and optionally a *UAS*-driven transgene. Shown is a schematic representation of a cross to the *40D*^{UAS} line described in this manuscript. Based on whether the F1 progeny of this cross shows a dominant phenotype(s), researchers can assess whether VDRC KK RNAi lines are suitable for their experiments and/or genetic screens.

SUPPLEMENTARY TABLE 1

	Transformant ID	Construct ID	Gene CG number	Gene symbol	pKC26 integrated at non-annotated pKC43 (30B)	pKC26 integrated at annotated pKC43 (40D)
1	104523	109756	*CG4005*	*yki*	x	x
2	106174	101055	*CG12072*	*wts*	x	x
3	101323	107562	*CG33193*	*sav*	x	x
4	107645	108458	*CG14217*	*Tao*	x	x
5	108254	107857	*CG17090*	*HipK*	x	x
6	105093	113571	*CG5651*	*pix*	x	x
7	104169	101704	*CG11228*	*hpo*	x	
8	106507	111409	*CG33967*	*kibra*	x	
9	109281	100573	*CG4114*	*ex*	x	
10	101497	108877	*CG8544*	*sd*	x	
11	108863	101190	*CG3352*	*fat*	x	
12	108260	108304	*CG11009*	*Wbp2*	x	

SUPPLEMENTARY TABLE 2

**Gal4 driver**	**Control**	***40D^{UAS}***
Actin	NP	pupal lethal
Tubulin	NP	pupal lethal
Eyeless	NP	NP
GMR	NP	NP
Dorsal	NP	smaller, held out wing
Patched	NP	smaller wings, held up and out
Fringe	NP	small, deformed, held back wing
Lozenge	NP	NP
Serrate	NP	smaller, held out wing
Hedgehog	NP	smaller, held out wing
Engrailed	NP	held back wing
Scalloped	NP	small, held out wing
Apterous	mildly curled wing	small, deformed wing
Rotund	NP	smaller, held out wing
MS1096	NP	small, deformed, held back wing
Nubbin	NP	small, uninflated wing
32B	NP	small, held back wing
71B	NP	smaller, held up wing
Spalt major	NP	smaller, held out wing
C5	NP	NP
Pannier	NP	NP
Myosin 1A	NP	NP

Twist	NP	NP
dNab	NP	NP
Elav	NP	NP

(NP: no phenotype)

SUPPLEMENTARY METHODS

Drosophila genetics

Male flies from the Bloomington TRiP and VDRC 'GD' RNAi collections, the VDRC genetic background (GD60100), *40D*^{UAS}, *w;;UAS-GFP* and *w;; UAS-tio*[2] strains were crossed to *w; GMR-Gal4, UAS-Yki*^{S168A}*-YFP/TM6B* [3], *w;; GMR-Gal4, UAS-EYFP/TM6B, w;; GMR-Gal4, UAS-Myc/TM3,* or *w; GMR-Gal4, UAS-InR/CyO* virgin female flies.

For the experiment described in Supplementary Figure 1f,g, genotypes were *w; GMR-Yki/+* and *w; GMR-Yki/+; MBDlike*^{1}. Flies carrying the *MBDlike*^{1} allele (*P{EPgy2}MBD-like*^{EY04582})[4] were from Bloomington, USA. Flies carrying the *GMR-Yki* transgene have been described in[5].

To generate the genotypes in Supplementary Figure 4 and Supplementary Table 2, we crossed *40D*^{UAS} or VDRC genetic background (GD60100) virgin females to males carrying the indicated Gal4 drivers.

At least 30 adult eyes of 1-2 day old females were assessed.

SUPPLEMENTARY REFERENCES

1 Green, E. W., Fedele, G., Giorgini, F. & Kyriacou, C. P. A Drosophila RNAi collection is subject to dominant phenotypic effects. *Nature methods* **11**, 222-223, doi:10.1038/nmeth.2856 (2014).

2 Denholm, B. *et al.* The tiptop/teashirt genes regulate cell differentiation and renal physiology in Drosophila. *Development* **140**, 1100-1110, doi:10.1242/dev.088989 (2013).

3 Poon, C. L., Lin, J. I., Zhang, X. & Harvey, K. F. The sterile 20-like kinase Tao-1 controls tissue growth by regulating the Salvador-Warts-Hippo pathway. *Developmental cell* **21**, 896-906, doi:10.1016/j.devcel.2011.09.012 (2011).

4 Marhold, J., Kramer, K., Kremmer, E. & Lyko, F. The Drosophila MBD2/3 protein mediates interactions between the MI-2 chromatin complex and CpT/A-methylated DNA. *Development* **131**, 6033-6039, doi:10.1242/dev.01531 (2004).

5 Huang, J., Wu, S., Barrera, J., Matthews, K. & Pan, D. The Hippo signaling pathway coordinately regulates cell proliferation and apoptosis by inactivating Yorkie, the Drosophila Homolog of YAP. *Cell* **122**, 421-434, doi:10.1016/j.cell.2005.06.007 (2005).
